# Serum adiponectin in Indian women with polycystic ovary syndrome

**DOI:** 10.6026/97320630019206

**Published:** 2023-02-28

**Authors:** Aishwarya Rajendra, Swathi Thilak, Veluri Ganesh

**Affiliations:** 1 Department of Obstetrics and Gynaecology, Mavintop Hospital and IVF Center, Davangere, Karntaka – 577004, India; 2 SS Institute of Medical Sciences, Davanagere, Karnataka, India; 3 Department of Biochemistry, Akash Institute of Medical Sciences and Research Centre, Bangalore-562110, India

**Keywords:** Adiponectin, Dyslipidemia, Poly Cystic Ovary Syndrome

## Abstract

Poly Cystic Ovary Syndrome (PCOS) is mainly characterised by reproductive hormonal imbalance and increased androgen levels, ovulatory dysfunction and polycystic appearing ovaries on ultrasonography. The obesity is common factor to cause PCOS and this adipocytokines play an important role to cause obesity related disorders. Hence, the present study aimed to evaluate the role of adiponectin in women with PCOS compared them with healthy controls. This case control study included total eighty (80) subjects out of this fourty subjects with PCOS and remaining fourty (40) age and body mass index matched healthy controls. The fasting, post parandial blood sugars, glycated haemoglobin, lipid profile and adiponectin levels were measured and data was recorded. There was a significantly elevated levels of total cholesterol, triacylglycerol, very low density lipoprotein, low density lipoprotein (p = 0.0001**) and there were no significant levels of fasting and post parandial blood sugars, glycated haemoglobin levels were observed in women with PCOS than in controls. Additionally, we also observed significantly decreased levels of high density lipoprotein and adiponectin in women with PCOS when compared to healthy controls (p = 0.0001**). The adiponectin shown a positive correlation with age, high density lipoprotein and negative correlation with total cholesterol, triacylglycerol, very low density lipoprotein and low density lipoprotein (p = 0.0001**). The altered adiponectin levels in polycystic ovary syndrome appear to form an important link between obesity and the complications of PCOS.

## Background:

Poly Cystic Ovary Syndrome (PCOS) is mainly characterised by reproductive hormonal imbalance and increased androgen levels, ovulatory dysfunction and polycystic appearing ovaries on ultrasonography [1]. However, the clinical presentation varies widely and often women with PCOS present for treatment of menstrual disturbances, hirsutism and infertility. Ethnicity is reported to influence the pattern of clinical presentation of PCOS. Disturbances in the menstrual cycles are one of the common features in women with PCOS [2]. Oligomenorrhea, amenorrhoea and prolonged menstrual bleeding are the common menstrual symptoms in these women. Oligomenorrhea is defined as less than 8 cycles per year or cycles longer than 35 days. Amenorrhea is the absence of menstruation for more than 3 months in the absence of pregnancy [3]. Oligomenorrhea is a more common feature observed in 85%-90% of women with PCOS; whereas about 30%-40% of women complaining of amenorrhea were found to have PCOS [4]. Normal menstruation is observed in about 30% of women with PCOS. The family history, obesity, epilepsy, type 1 diabetes mellitus, type 2 diabetes mellitus, gestational diabetes mellitus were the other conditions reported to be associated with an increased risk of PCOS [5]. The Rotterdam consensus workshop included polycystic ovaries as one of the criteria for diagnosing PCOS. The consensus defined polycystic ovaries as having 12 or more follicles, measuring between 2 and 9 mm in diameter and/or an ovarian volume > 10 cm3 [6]. However the presence of polycystic ovaries alone is not sufficient to diagnose PCOS since 20%-30% of normal women have evidence of presence of multiple cysts in the ovaries. Further advancements in ultrasound imaging technique have resulted in a better definition of polycystic morphology [7]. Obesity is recognised as one of the common features of polycystic ovary syndrome; however it is not essential for the diagnosis. A high prevalence of obesity and overweight has been reported in western as well as Indian women with PCOS [8]. In addition to being associated with the condition, obesity amplifies the metabolic and endocrine consequences of PCOS. The occurrence of obesity in PCOS women is influenced by various factors including genetic and environmental factors such as lack of physical exercise, intake of high calorie diet [9]. Adiponectin is an important protein synthesised and secreted from adipose tissue. Adipose tissue was traditionally considered as an inert organ that is involved in storage of fat [10]. However, since recent times, adipose tissue is regarded as a metabolically active organ, participating in various metabolic functions through release of several locally and systemically biologically active molecules known as adipokines [11]. The role of adipocytokines, particularly adiponectin has been implicated in the pathophysiology of PCOS. In case of healthy individuals, adiponectin levels were found to correlate inversely with the degree of adiposity and were also found to be influenced by the degree of insulin resistance and hyperinsulinemia [12]. Studies have shown that circulating adiponectin levels were lower in women with PCOS and contribute to the complications associated with PCOS. The altered levels may occur as a result of increased obesity or insulin resistance that are commonly observed in these women or independent of these factors [13]. The decreased adiponectin levels may further cause decreased insulin sensitivity leading to insulin resistance, thus resulting in the development of a vicious circle [14]. The altered adiponectin levels are proposed to further contribute to the endocrine and metabolic disturbances observed in PCOS women. Based on this background the present study aimed to determine the role of adiponectin in women with PCOS.

## Materials and Methods:

The present study included a total of fourty (40) women attending "District hospital, Haveri, Karnataka" and diagnosed with polycystic ovary syndrome based on Rotterdam criteria. Fourty (40) age matched healthy women were recruited as controls. All the participants were included after an informed consent. The study was approved by the institutional ethics committee. The subjects with diabetes, hypertension, renal, liver, thyroid disease, cardio vascular disease and acute infections excluded from this study.

## Collection of samples and methods:

Five (5) mL of fasting venous blood sample was collected from all the subjects into two tubes: 1mL into a tube containing anticoagulant, and 4 mL into a plain tube. Plasma samples were separated immediately and plain samples were allowed to clot and separated by centrifugation at 3000 rpm for 15min. The separated samples were transferred into appropriately labeled aliquots and stored at -80°C until biochemical analysis was done. Fasting and post parandial blood sugars were analysed by glucose oxidase peroxidase (GOD-POD) method, glycated haemoglobin was measured by latex immuno turbidimetric method, lipid profile was analysed by laboratory standard methods and adiponectin were estimated by Enzyme Linked Immunosorbent Assay (ELISA kit obtained from Biocodon Technologies, Kanasas, USA).

## Statistical Analysis:

Continuous variables were expressed as mean ±plusmn; SD. The Kolmogorov-Smirnov test was used to evaluate the distribution of continuous variables. Data which were not normally distributed were expressed as or median (interquartile range). The biochemical parameters studied in patients and controls were compared using unpaired two tailed Student's t-test or Mann Whitney U test as appropriate. The association between the variables was studied using Pearson or Spearman correlation analysis. A p value<0.05 was considered as statistically significant. Statistical analysis was done using Microsoft excel spread sheets and SPSS version 16.0.

Table 1 shows the anthropometric and biochemical parameters studied in healthy controls and PCOS women. Both groups were matched with respect to age, PCOS women were obese when compared to healthy women (p = 0.0001**). The fasting and post parandial blood sugars and HbA1c levels were not significant between the PCOS Women and controls. PCOS women had significantly higher total cholesterol, triglycerides, very low density lipoprotein and low density lipoprotein when compared to controls respectively P = 0.0001**. There was a significantly decreased levels of serum adiponectin concentration was found in PCOS women when compared to healthy controls (p=0.0001**).

The correlation of adiponectin with the parameters studied using Pearson correlation analysis is presented in table-2. Adiponectin showed a significant positive correlation with age, high density lipoprotein (p= 0.05* and 0.0001**) and negative correlation with body mass index, total cholesterol, triacylglycerol, very low density lipoprotein and low density lipoprotein (p=0.0001**). Additionally, we also observed there were no significant correlation between adiponectin and fasting blood sugars, post parandial blood sugars and glycated haemoglobin (p=0.0289, 0.071 and 0.090).

##  Discussion:

Polycystic ovary syndrome (PCOS) is one of the most common endocrine-metabolic disorders of women of reproductive age group. PCOS occurs as a result of interaction between environmental factors and intrinsic factors and begins to appear early in the reproductive age. Insulin resistance, which is commonly observed in PCOS women, is another contributing factor for PCOS [15]. PCOS is characterized by a clustering of chronic anovulation, hyperandrogenemia and polycystic appearing ovaries on ultrasound. Women with PCOS often seek treatment for menstrual disturbances, hirsutism and infertility [16]. In addition to the hormonal changes, metabolic disturbances including glucose intolerance and dyslipidemia are also a common feature of polycystic ovary syndrome. Obesity, which is observed in more than half of women with PCOS, plays an important role in the metabolic complications associated with PCOS [17]. Adiponectin is an important adipocytokine synthesized and secreted by white adipocytes. Adiponectin exerts insulin sensitizing, anti-inflammatory, vasoprotective, antiapoptotic and anti-atherogenic effects [18]. Though mainly produced by the adipose tissue, circulating adiponectin levels were found to paradoxically decrease in obese individuals and this decrease in adiponectin levels has been proposed to contribute to the complications associated with obesity [19]. Several studies have evaluated the relationship between PCOS and adiponectin. Majority of studies have reported significantly lower adiponectin levels in PCOS women when compared to BMI matched healthy controls [20]. In the present study, PCOS women were overweight and had significantly higher BMI than controls (5.96 ± 1.94 Vs 16.10 ± 4.23, p = 0.0001**) (Table-1). Several factors have been proposed to be responsible for the lower adiponectin levels in PCOS women. While some of the studies have suggested that the alterations in adiponectin levels are a result of insulin resistance and glucose intolerance (37, 38), others have shown that adiponectin concentration varies with the degree of adiposity and is not influenced by insulin resistance [21]. Another recent study has reported that hypoadiponectinaemia is present in both obese and lean women with PCOS with variable degrees of insulin resistance. On the other hand, other recent studies who have also observed decreased total adiponectin and HMW adiponectin levels in PCOS women suggested that the lowered adiponectin levels occur independent of BMI and insulin resistance and that the posttranscriptional/translational modifications contribute to the low levels of HMW adiponectin in PCOS [22]. Examination of adipose tissue in women with PCOS has shown that the expression of messenger RNA (mRNA) for adiponectin is significantly lower in women with PCOS compared with weight-matched women without PCOS. This decreased expression of adiponectin mRNA, which was observed in both subcutaneous and visceral fat tissue, was found to be consistent with the lower levels of circulating adiponectin levels that are seen in women with PCOS [23]. The increased adiposity as indicated by a higher BMI in women with polycystic ovary syndrome in the present study might be responsible for the lower adiponectin levels observed in them. However, we have reported higher adiponectin levels in young non-obese women newly diagnosed with PCOS when compared to controls. Altered adiponectin levels have been reported in relation to lipid disturbances in PCOS women. In the present study, serum lipid profile was measured in PCOS women and compared with healthy controls. PCOS women had significantly higher total cholesterol, triglyceride, very low density lipoprotein and low density lipoprotein levels when compared to controls respectively (p=0.0001**) (Table 1). However, the HDL cholesterol levels were reduced in PCOS women when compared with controls (p=0.0001**) (Table 1). Dyslipidemia is commonly observed in PCOS women and contributes to the increased risk of metabolic syndrome and cardiovascular disease in these women. Earlier studies have reported an increased total cholesterol and triglycerides and decreased HDL cholesterol in PCOS women, compared to controls [24]. The dyslipidemia in the setting of polycystic ovary syndrome can occur due to multiple causes. The increased prevalence of obesity, insulin resistance and hyper androgenemia have all been proposed to be involved in the lipoprotein disturbances observed in PCOS women [25]. Increased lipogenesis, decreased clearance, reduced oxidation of fatty acids and their increased availability and an increased secretion of very low density lipoprotein (VLDL) particles by the hepatocytes contribute to the increased triglyceride levels in the presence of insulin resistance [26]. The association of adiponectin with the clinical and biochemical parameters was analysed using Pearson correlation analyses. The adiponectin showed a significant positive correlation with age, high density lipoprotein and significant negative correlation with body mass index, total cholesterol, triacylglycerol, very low density lipoprotein and low density lipoprotein respectively (p = 0.00001**) (Table 2). Another recent study observed significant inverse correlation between adiponectin and age in PCOS women, whereas similarly another study on sixty women with PCOS has reported that adiponectin was associated with obesity. Further, adiponectin levels in PCOS women in the present study showed non-significant correlation with fasting and post parandial blood sugars and glycated haemoglobin (Table 2). However, another recent study has reported that adiponectin showed significant negative correlation with insulin resistance [27]. The discrepancy in the results could be due to a small sample size and the difference in the study groups. Thus, findings of the present study indicate that serum adiponectin levels are significantly lower in women with polycystic ovary syndrome compared to healthy women. The hyperandrogenemia that is one of the characteristic features of PCOS leads to a state of adiposity which further results in decreased adiponectin levels. The low adiponectin levels further contribute to the metabolic complications associated with PCOS including insulin resistance and dyslipidemia.

## Conclusion:

Thus, the altered adiponectin levels in polycystic ovary syndrome appear to form an important link between obesity and the complications of PCOS. Therapeutic interventions using drugs such as metformin and weight reduction programmes are known to improve adiponectin levels and may provide beneficial effects. Thus, though adiponectin is found to be involved in the hormonal and metabolic disturbances of PCOS.

## Figures and Tables

**Table 1:** Anthropometric and biochemical parameters among the study subjects

**Parameters**	**Controls**			**PCOS Patients**			**P-Values**
Age	34.82	±	4.63	37.53	±	5.16	0.031*
BMI	22.34	±	2.03	38.68	±	5.72	0.0001**
FBS	90.62	±	3.68	93.81	±	7.64	0.630 10186
PPBS	129.46	±	6.34	132.09	±	4.65	0.53110186
HbA1c	4.62	±	1.35	5.43	±	1.91	0.886 10186
TC	163.24	±	21.03	223.61	±	44.32	0.0001**
TAG	92.22	±	14.51	183.26	±	32.17	0.0001**
HDL	42.16	±	17.48	39.17	±	12.36	0.0001**
VLDL	25.78	±	4.55	42.51	±	8.72	0.0001**
LDL	69.16	±	5.77	135.62	±	17.55	0.0001**
Adiponectin	16.1	±	4.23	5.96	±	1.94	0.0001**
Data expressed as mean±plusmn;SD, *median (interquartile range, IQR),p value obtained using student t test or Mann Whitney U test, as appropriate, P: Probability, 10186: Not Significant, *: Significant, **: Highly significant, PCOS: polycystic ovary syndrome; BMI: body mass index; FBS: fasting blood sugar; PPBS: Post Parandial Blood Sugars, HbA1c: Glycated Haemoglobin, TC: total cholesterol; TGL: triglycerides; VLDL: Very Low Density Lipoprotein, LDL: Low Density Lipoprotein, HDL-C: high density lipoprotein cholesterol.

**Table 2:** Correlation of adiponectin with other parameters in women with PCOS

**Adiponectin**		
**Parameters **	**r **	**P- Values **
Age	0.628	0.05*
BMI	-0.465	0.0001**
FBS	0.344	0.289 10186
PPBS	0.204	0.071 10186
HbA1c	0.699	0.090 10186
TC	-0.323	0.0001**
TAG	-0.982	0.0001**
HDL	0.475	0.0001**
VLDL	-0.23	0.0001**
LDL	-0.856	0.0001**
r: Correlation Coefficient, P: Probability, 10186: Not Significant, *: Significant, **: Highly significant, PCOS: polycystic ovary syndrome; BMI: body mass index; FBS: fasting blood sugar; PPBS: Post Parandial Blood Sugars, HbA1c: Glycated Haemoglobin, TC: total cholesterol; TGL: triglycerides; VLDL: Very Low Density Lipoprotein, LDL: Low Density Lipoprotein, HDL-C: high density lipoprotein cholesterol.
